# ICOS and OX40 tandem co-stimulation enhances CAR T-cell cytotoxicity and promotes T-cell persistence phenotype

**DOI:** 10.3389/fonc.2023.1200914

**Published:** 2023-08-18

**Authors:** Eider Moreno-Cortes, Pedro Franco-Fuquen, Juan E. Garcia-Robledo, Jose Forero, Natalie Booth, Januario E. Castro

**Affiliations:** ^1^ Division of Hematology and Medical Oncology, Mayo Clinic, Phoenix, AZ, United States; ^2^ Cancer Research and Cellular Therapy Laboratory, Mayo Clinic, Phoenix, AZ, United States; ^3^ Division of Internal Medicine, The Ohio State University Wexner Medical Center, Columbus, OH, United States; ^4^ Center for Cancer and Blood Disorders, Phoenix Children’s Hospital, Phoenix, AZ, United States

**Keywords:** adoptive immunotherapy, chimeric antigen receptor, cell-and tissue-based therapy, T-cell activation, costimulatory molecules

## Abstract

Chimeric Antigen Receptor (CAR) T-cell therapies have emerged as an effective and potentially curative immunotherapy for patients with relapsed or refractory malignancies. Treatment with CD19 CAR T-cells has shown unprecedented results in hematological malignancies, including heavily refractory leukemia, lymphoma, and myeloma cases. Despite these encouraging results, CAR T-cell therapy faces limitations, including the lack of long-term responses in nearly 50-70% of the treated patients and low efficacy in solid tumors. Among other reasons, these restrictions are related to the lack of targetable tumor-associated antigens, limitations on the CAR design and interactions with the tumor microenvironment (TME), as well as short-term CAR T-cell persistence. Because of these reasons, we developed and tested a chimeric antigen receptor (CAR) construct with an anti-ROR1 single-chain variable-fragment cassette connected to CD3ζ by second and third-generation intracellular signaling domains including 4-1BB, CD28/4-1BB, ICOS/4-1BB or ICOS/OX40. We observed that after several successive tumor-cell *in vitro* challenges, ROR1.ICOS.OX40ζ continued to proliferate, produce pro-inflammatory cytokines, and induce cytotoxicity against ROR1^+^ cell lines *in vitro* with enhanced potency. Additionally, *in vivo* ROR1.ICOS.OX40ζ T-cells showed anti-lymphoma activity, a long-lasting central memory phenotype, improved overall survival, and evidence of long-term CAR T-cell persistence. We conclude that anti-ROR1 CAR T-cells that are activated by ICOS.OX40 tandem co-stimulation show *in vitro* and *in vivo* enhanced targeted cytotoxicity associated with a phenotype that promotes T-cell persistence.

## 1 Introduction

Chimeric antigen receptor (CAR) T-cell therapy has revolutionized cancer treatment by redirecting the immune system to specifically target and eliminate cancer cells (1). Despite its success in treating hematological malignancies, CAR T-cell therapy is not entirely effective in approximately 50-70% of patients with leukemia, lymphoma, or myeloma (1–3). Additionally, the results of clinical trials in solid tumors have been somewhat disappointing (1, 4–6). The reasons for CAR T-cell therapy’s failure are complex and include the immunosuppressive effect of the tumor microenvironment (TME) and the low expansion and short persistence of the immune effector T-cells (1, 4)

In patients with hematologic malignancies, long-lasting clinical remissions are associated with robust expansion and persistence of genetically modified CAR T-cells (7, 8). Lack of CAR T-cell persistence has been shown consistently in clinical trials as one of the most critical factors associated with poor overall efficacy (9–11). Similarly, successful treatment of solid tumor patients requires vigorous expansion and long term-persistence of CAR T-cells (12).

CAR T-cell persistence depends on various factors, including the patient/donor immunosuppressed status, laboratory culture conditions of the engineered T-cells, T-cell exhaustion and metabolic pathways involved, a disproportionately high ratio of effector memory *vs*. central memory T-cells, and host immune responses (10, 11, 13, 14). However, one of the modifiable variables that can improve CAR T-cell persistence is the CAR construct design, particularly the selection and optimization of intracellular signaling domains (ICD) (15–17). Therefore, substantial research efforts are centered on the molecular architecture of the CAR construct as it may significantly impact T-cell expansion, persistence, and clinical outcomes (18–20).

Most transmembrane CAR modules come from molecules like CD8 and CD28, essential for T-cell function (21, 22). The CD3ζ chain and other costimulatory domains linked in cis are placed in the intracellular module (23). The T cell receptor CD3ζ chain and one or more signaling domains from the costimulatory proteins CD28, 4-1BB, OX40, CD27, or ICOS typically comprise the ICD (18). The CARs that are currently FDA approved, and the majority of products undergoing clinical trial testing use either CD28 or 4-1BB ICD linked to CD3ζ (Second generation – 2G CARs) (5, 24). Novel tandem ICDs composed of three domains, such as CD28 and 4-1BB ICD followed by CD3ζ (Third generation – 3G CARs), are gathering significant interest based on impressive preliminary results (1, 5, 25–29).

The goal of incorporating different ICDs is to enhance the complementary features of each domain and generate a CAR signaling component with improved features. For example, CD28 and 4-1BB induce intracellular signaling through different pathways. CD28 is a powerful early stimulator, whereas 4-1BB is more critical in the later activation of cellular expansion phases, particularly memory T-cells (30). Moreover, experimental studies have shown that T cells stimulated with tandem CD28 and 4-1BB ICDs (3G-CARs) have greater intracellular signaling activation, more potent antitumor activity, and longer *in vivo* persistence than T cells stimulated by 2G CARs (31).

Because CAR construct design is critical for the development of highly efficient CAR T-cells with optimal expansion and persistence, particularly for applications in solid tumors (8, 32–34), we sought to evaluate the novel combination of ICOS (Inducible T-cell co-stimulator) and OX40 (CD134) used as tandem ICD in combination with CD3ζ (3G-design) and studied their effect *in vitro* and *in vivo*. Our model uses CAR T-cells redirected against ROR1, a promising tumor-associated antigen (TAA) with a broad expression range in hematological malignancies and solid tumors (35–39). We selected to study ICD derived from ICOS and OX40 because of their potential role in CAR T-cell function and persistence. ICOS and OX40 are costimulatory molecules involved in the regulation and expression of anti-apoptotic molecules such as Bcl-2, and Bcl-xL in T-cells, increase secretion of IL-2, IL-21, and enhance the expression of costimulatory molecules like CD40-L (CD154) and CD127 that regulate T cell survival and function (20, 32, 40–44). In addition, ICOS and OX40 can enhance the expansion of central memory T-cells and induce Th1 and Th17 polarization required to maintain long-lived persistent T-cells with the ability to develop immunity against previously encountered antigens (32, 41, 42).

## 2 Materials and methods

### 2.1 Cell lines

The ROR1^+^ JeKo-1 (Mantle cell lymphoma) and ROR1^-^ K562 (Chronic myelogenous leukemia) Cell lines were initially obtained from ATCC; cell lines were obtained more than six months before experiments, and authentication was performed by cell banks utilizing short tandem repeat profiling. All cell lines were tested for mycoplasma contamination (MycoAlert Mycoplasma Detection Kit, LT07-318, Lonza). The number of passages was limited to 10. These cell lines were transduced with a luciferase-ZsGreen lentivirus (Addgene) and sorted with FACS Aria II (B.D. Biosciences) instrument to 100% purity. Cell lines K562 were used as controls, as indicated in the relevant figures. The cell lines were maintained in culture with RPMI1640 (Gibco) supplemented with 10% Fetal bovine serum (Gibco) and 50 U/mL penicillin/streptomycin (Gibco, Life Technologies, 15070–063). Primary cells were thawed at least 12 hours before the experiment for all functional studies and rested at 37°C. The use of recombinant DNA was approved by the Institutional Biosafety Committee (IBC HIP00000710.04).

### 2.2 Vector design

Second-generation and third-generation ROR1 CAR constructs were synthesized *de novo* (GenScript). All the constructs consist of a scFv (single-chain variable fragment) against ROR1 (Clone R12) linked with an IgG4 hinge (Uniprot P01861, section 99-110) cloned into a second-generation 4-1BB and third-generation CD28 + 4-1BB, ICOS + 4-1BB, and ICOS + OX40 costimulated CAR in a third-generation lentivirus (**Table 1**). Third-generation Lentiviral vectors were generated using transient transfection of the plasmid into 293T cells obtained from ATCC in the presence of Lipofectamine 2000 (Invitrogen), VSV-G, RSV-Rev, pMDLg-pRRE (Adgene).

**Table 1:** Intracellular Costimulatory domains (ICD).

Costimulatory molecule (ICD)	CAR T-cells – Functional benefit
4-1BB (CD137)	Stimulates CD8^+^ central memory T cell generation. Favors CAR T-cell persistence.
CD28	Potent cytotoxic function; IL-2 production; may favor CD4^+^ T cell expansion
ICOS (CD278)	Enhances Th1 and Th17 polarization. Favors CAR T-cell persistence
OX40 (CD134)	Suppresses Treg development
3G-Tandem ICDs	CAR T-cells – Functional benefit
CD28 + 4-1BB	Enhanced antitumor effect and ameliorates proliferative capacity, retention of a memory phenotype, and reduced exhaustion.
CD28 + OX40	Sustained clonal expansion
ICOS + 4-1BB	Enhanced antitumor effects and increased persistence *in vivo*, including solid tumor models.
OX40 + 4-1BB	Favors CAR T-cell persistence; may favor CD8^+^ T cell expansion
ICOS + OX40	Expected enhanced antitumor effect and increased persistence. Abrogates IL10 and Treg development.

Functional T cell function of each intracellular costimulatory domain (ICD) molecule tested in this study with a combinatory effect hypothesis for third generation constructs.

### 2.3 CAR-T cell generation

Briefly, peripheral blood mononuclear cells (PBMC) are isolated from de-identified healthy donor blood apheresis samples (Vitalant). T-cell isolation from previously isolated PBMCs was performed using an 8-minutes cell isolation kit immunomagnetic negative selection (STEMCELL magnetic beads against CD19, CD16, CD15, CD14, CD34, CD56, CD123, and CD235a), obtaining a purity after isolation greater than 95% of the T-cell product. Primary cells were cultured in T-cell medium (TCM) supplemented with OpTmizer CTS, 1% penicillin-streptomycin-glutamine (Gibco), and IL-2 at a concentration of 100 U/mL (PeproTech).

Previously isolated T cells were stimulated using a Dynabeads CD3/CD28 generated in-house at a 1:3 cell: bead ratio. After 24 hours of stimulation, T cells were transduced with lentiviral particles at an MOI (multiplicity of infection) of 3.0. Beads were removed from the T cell expansion using DynaMag-50 (Invitrogen) on day 6. The expression of CAR was analyzed by flow cytometry on day 7. CARs were stained with a biotinylated ROR1 protein followed by a PR-conjugated streptavidin. On day 12, CART- Cells were harvested and cryopreserved in freezing medium made from 90% FBS (Gibco) and 10% DMSO (Millipore Sigma) for planned experiments. The CAR T cells were thawed and left resting in TCM for at least 12 hours before individual experiments.

### 2.4 Phenotypic and exhaustion analysis

All experiments were performed on a FACS Symphony (B.D. Biosciences), and data obtained were analyzed with FlowJo software (version 10.7.1). CAR expression was evaluated using a ROR1 biotinylated protein (ACRO Biosystem) and a streptavidin (PE-conjugated; B.D. biosciences). T cell profile and characterization were evaluated using mAb to CD45, CD3, CD4, CD8, CD62L, CCR7, PD-1, TIM-3, LAG-3, TIGIT, and the corresponding isotype controls (B.D. Biosciences). The presented results presenting CAR T cell phenotype data were performed using the following gating strategy: we excluded the doubles, by side scatter versus forward scatter, viability using LIVE/DEAD Fixable Aqua Dead Cell Stain Kit (Invitrogen), followed by the populations CD45^+^, CD3^+^, CAR^+^ Cells (**Supplementary Figure 1**).

### 2.5 Multiparametric flow cytometry

Anti-human and anti-mouse antibodies were purchased from BioLegend, eBioscience, or B.D. Biosciences. BD FACS lyse buffer (B.D. Biosciences) was used to lyse mouse red blood cells (JeKo-1 xenograft) peripheral blood samples (day −1 and 8 of ROR1 CART-cell infusion) before staining and flow cytometric analysis. Cells from *in vitro* culture (*in vitro* antigen-specific degranulation/cytokine production assays and antigen-specific proliferation assay). Before staining, cells were washed twice in a flow buffer [PBS supplemented with 1% FBS and 1% sodium azide (Ricca Chemical)] and stained at room temperature unless specified for the antibody. For cell number quantitation, Countbright beads (Invitrogen) were used according to the manufacturer’s instructions (Invitrogen). In all analyses, the population of interest was gated based on forward versus side scatter characteristics, followed by singlet gating, and live cells were gated following staining with LIVE/DEAD Fixable Aqua Dead Cell Stain Kit (Invitrogen).

Surface expression of the CAR was detected by staining with a ROR1 biotinylated protein. In brief, an aliquot of the CAR-T cells (e.g., 50,000 T cells) was first washed and then resuspended in 50 μL of a flow buffer. Cells were stained with 1 μL of ROR1 biotinylated protein and 0.3 μL of LIVE/DEAD Fixable Aqua Dead Cell Stain Kit to exclude dead cells and incubated in the dark for 15 minutes at room temperature. After incubation, cells are washed by adding 150 μL of a flow buffer and centrifuged at 650 × g for 3 minutes at 4°C. Then 8 μL of streptavidin-PE and 50 μL of flow buffer were added, and cells were incubated for 30 minutes at 4°C. After surface staining, cells are fixed and permeabilized by adding 100 μL of a fixation medium and incubated for 15 minutes at room temperature. Cells are then washed with 100 μL of a flow buffer. Fixed/permeabilized cells were resuspended in 50 μL of a permeabilizing buffer. Cells were washed and resuspended in 200 μL of a flow buffer and acquired on a flow cytometer. Flow cytometry was performed on a Symphony (B.D. Biosciences) five-laser cytometer (**Supplementary Table 1**). Analyses were performed using FlowJo X10.0.7r2 software.

### 2.6 Cytokine release assay

IFN-γ and CD107a production were evaluated using flow cytometry, according to the manufacturer’s instructions. Briefly, 0.5x10^6^ CAR T cells were seeded for (IFN-γ 12 hrs, CD107a 4 hrs) with 5x10^5^ target cells (Jeko-1 or K562). To create stringent testing conditions similar to physiological environment we tested an E:T ratio of 1:1, in triplicate wells on 96-well round bottom plates as reported in previous studies (45–47). Negative and positive controls were represented by CAR T cells that remained unstimulated (medium only) or treated with 40 ng/mL of PMA and 4 mg/mL of ionomycin (Sigma-Aldrich). After the stimulation, the corresponding antigens were measured using FACS in the Flow Cytometry core at Mayo Clinic AZ.

### 2.7 *In vitro* cytotoxicity and rechallenging assay

We developed a Luciferase-based rechallenge cytotoxicity assay. The target Luciferase^+^ Jeko-1 and K562 cells were incubated with effector CAR T-cells transduced with different constructs as indicated. Each day the E:T ratios were adjusted to generate seven different E:T ratios per construct. Effector cells remain unchanged from day one until day five to assess their ability to induce cytotoxicity during this rechallenge experiment. On the other hand, fresh new target cells were added as needed to adjust the E:T ratios. Each day and for each construct, we measured the cytotoxicity generated by the seven different E:T ratios, and with these data, we obtained the logarithmic trendline and calculated the E:T ratio that induced cytotoxicity in 50% of the target cells (EC50). The EC50 E:T ratio was used as a proxy for each cellular product’s potency and ability to induce repetitive cytotoxicity and expand over time (persistence). CAR T-cells transduced with constructs that induce low EC50 E:T ratios at five days are expected to be fitter and have a better persistence profile, while those with high EC50 E:T ratios are more prone to exhaustion. The killing was calculated by BLI on a Xenogen IVIS-200 Spectrum camera (catalog no. 124262, PerkinElmer) as a measure of residual live cells. Ten minutes before imaging, samples were treated with 1 mL D-luciferin (30 mg/mL, Gold Biotechnology) per 100 mL sample volume.

### 2.8 Evaluation of CAR T-cell tonic signaling (CD3z phosphorylation)

Tonic signaling evaluation using CD3z Pho(Tyr142) on ROR1 CAR constructs was performed. Different CAR T cells were expanded for 12 days with T cell media +100 U/ml of IL-2. The samples were electrophoresed using 4-12% Bis-Tris Protein Gel (Cat.NP0322BOX). Resolved proteins were transferred onto a nitrocellulose membrane (Cat.IB23001) by iBlot^®^ 2 Dry Blotting System (Cat. IB21001). The blot was probed with Phospho-CD3z (Tyr1 42) Polyclonal Antibody (Cat.PA5-37512, 1:1000 dilution) and Goat anti-Rabbit IgG (H+ L) Supercional™’ Recombinant Secondary Antibody, HRP (Cat. A27036, 1:20,000) using the Bright™M FL 1500 (Cat. A44115). Chemiluminescent detection was performed using SuperSignal™M West Alto Ultimate Sensitivity Substrate (Cat. A38556). The fact that CD3z was not present in the untransduced, and fresh T-cells confirms that the antibody is specific to native CD3z and CAR CD3z on controls.

### 2.9 *In vivo* CAR T-cell cytotoxicity assessment

The Mayo Clinic Hospital Institutional Animal Care and Use Committee approved all animal experiments. NOD scid gamma (NSG) mice were purchased from The Jackson Laboratory and housed in the Mayo Clinic, Arizona vivarium, under specific pathogen-free conditions in microisolator cages and were provided *ad libitum* access to autoclaved food and acidified water.

Male and female, 8 to 12-week-old, non-obese diabetic/severe combined immunodeficient bearing a targeted mutation in the IL2 receptor gamma chain gene (NSG) mice were purchased from the Jackson Laboratory (catalog no. 005557) and maintained within the Mayo Clinic Department of Comparative Medicine under an Institutional Animal Care and Use Committee–approved protocol (IACUC A00005886-21). The ROR1+luciferase+ MCL JeKo-1 cell line was used to establish a systemic ROR1+ tumor model. 5 × 10^5^ cells were resuspended in PBS and injected via the tail vein, 1 week before T-cell infusion. The tumor burden was assessed weekly by BLI using a Xenogen IVIS-200 Spectrum camera to confirm the engraftment of JeKo-1 cells and visual inspection. The mice were randomized according to the tumor burden assessed by IVIS to receive 3 × 10^6^ of (i) UTD, (ii) ROR1.BBζ, (iii) ROR1.IOζ or (iv) CD19.BBζ CAR T-cells (*Cells infused were normalized by CAR% of each group, except for the UTD group*). One week after T-cell infusion, mice were imaged with BLI, as described below. Mice were euthanized after the completion of the experiment.

### 2.10 Statistical analysis

A Student’s t-test was used to compare two value sets, while we used one-way ANOVA when three groups were involved. Histograms represent mean values ± standard deviations. P < 0.05, P < 0.01, or P < 0.001 were indicated by *, ** or ***, respectively. All statistics were performed using GraphPad Prism version 8.05 for Windows (GraphPad Software, www.graphpad.com). Statistical tests are described in detail in the representative figure legends.

## 3 Results

### 3.1 scFv optimization and functionality of ROR1 redirected CAR T-cell

**Figure 1:**
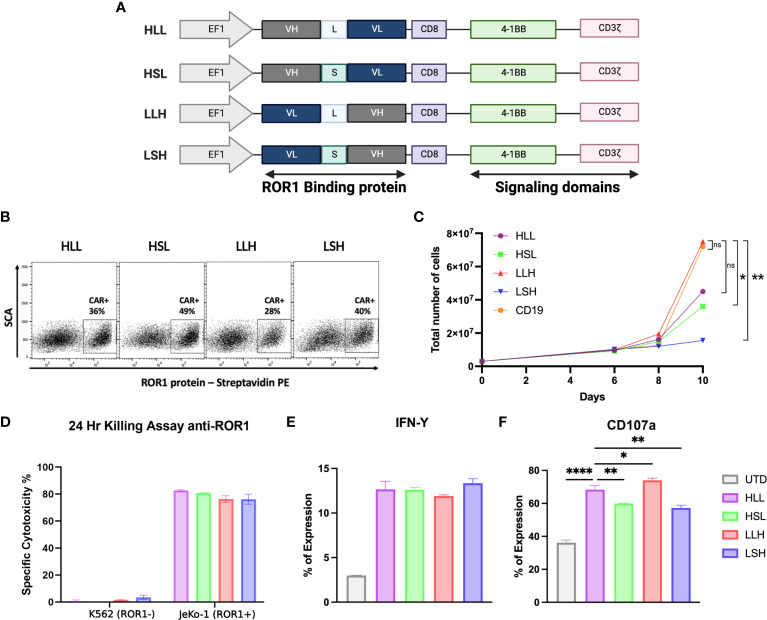
ScFv optimization. **(A)** Schematic diagram showing the different anti-ROR1 Sc variants used for testing, including four different permutations of the fusion proteins Heavy (VH) and light (VL) chains with two different (G4s)_3_ linkers - Long (L) and short (S). **(B)** Surface expression of anti-ROR1 specific CAR protein in primary T cells.1^6^ T cells were transduced with the different constructs using a lentiviral concentrate at a MOl 3.0. Expression was examined by flow cytometry at 6 days following transduction, using a ROR1-biotin soluble recombinant protein and streptavidin-PE. **(C)** CAR T cell expansion curve **(D)**. After 14 days of expansion using a luciferase-based assay the different scFv configurations, the constructs were incubated with JeKo-1/ROR1+ and K662/ROR1- cells at an ET ratio of 10:1, and the cells were harvested at 24 hours. **(E)** After 24 hours stimulation with ROR1+ cells IFN- γ was measure by FACS. **(F)** Degranulation assay after 4 hours simulation with ROR1+ cells. Data are plotted as mean ‡ SEM (****, p < 0.00001, **, p < 0.001, and * p= < 0.01).

We evaluated and optimized different scFv sequences derived from anti-ROR1 antibodies using a 2G-CAR construct design with 41BB-ζ ICD (**Figure 1A**) (48). First, we studied the effects of the cloning position of the variable heavy (VH) and variable light (VL) domains in the lentiviral plasmid vector and the effects of the interspace linker (GGGGS)n length in between the VH and VL domains - (short linker n=5 *vs*. long linker n=15). Specifically, we evaluated differences in the CARs’ ability to induce CAR expression after T-cell transduction, T-cell expansion, cytotoxicity, T-cell activation, or degranulation.

We observed that longer linkers induced lower levels of CAR expression, but these differences were not statistically significant (**Figure 1B**). Also, CAR constructs with long linkers, particularly the VL-long linker-VH (LLH) sequence, induced higher levels of T-cell expansion, reaching a 25-fold increase at day ten compared to the other constructs (**Figure 1C**).

All the ROR1-CAR constructs we tested induced similar cytotoxicity and IFN-γ production levels specifically driven by ROR1 expression on the target cells (**Figure 1D, E**). However, the level of degranulation measured by CD107a expression was higher on the T-cells transduced with long linker constructs (HLL, LLH) (**Figure 1F**).

### 3.2 3G ROR1 CAR constructs showed increased *in vitro* INF-γ secretion, long-term cytotoxicity, and increased T_CM_/T_EM_ persistence-associated phenotype

**Figure 2:**
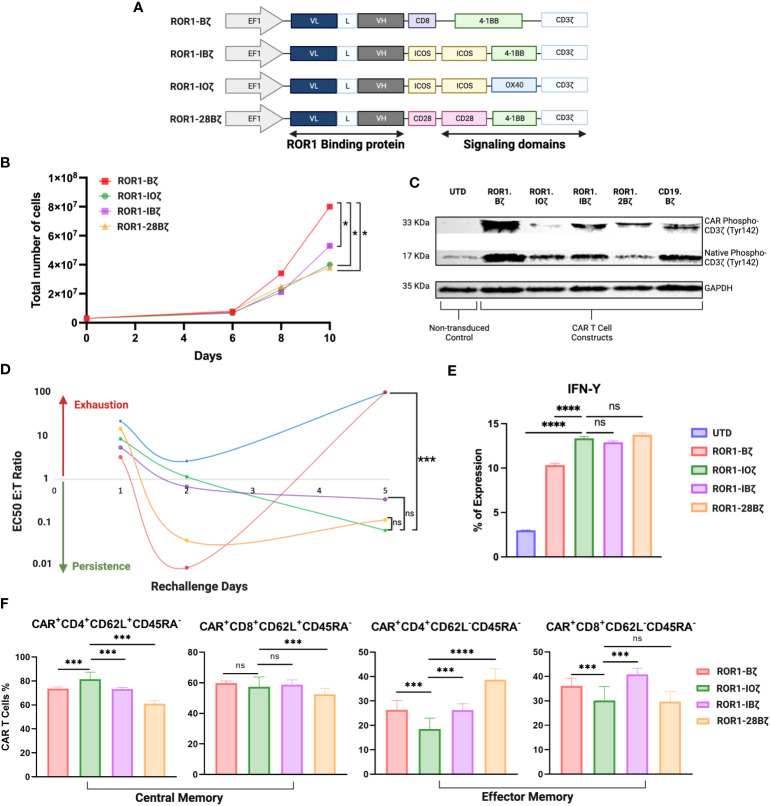
Third generation optimization. **(A)** Schematic diagram showing the different anti-ROR1 third generation constructs using the orientation light chain (VL) + long linker (G4s)_3_ + heavy chain (VH) ScFv using combination of the following co-stimulatory domains ICOS, OX40, 41BB and CD28. **(B)** Expansion curve of the different constructs. **(C)** Western blot of CART cell constructs tonic signaling evaluation after expansion measuring the phosphorylation of tyr142 CD3z **(D)**. After 14 days of expansion using a luciferase assay base the different second and third generations constructs were incubated with JeKo-1/ROR1* cells at an ET ratio of 10:1, with different re-challenges using the same E:T ratio of target cells for day 1, 2, 3, 4 and 5. **(E)** IFN- γ expression measured by FACS after 5 days of re-challenge **(F)**. Phenotype of CAR T cells as demonstrated by percentage of central memory (CD62L+CD45RA-) and effector memory (CD62L-CD45RA- CAR T cells 5 days after ROR1+ target cells stimulation every day. Data are plotted as mean ‡ SEM (****, p < 0.00001, ***, p < 0.0001, **, p < 0.001, and * p = < 0.01, ns: no statistically significant).

The above data suggest that the cloning positioning of VH or VL was not relevant to the CAR T-cell parameters that we measured, that induced cytotoxicity was similar in all the 2G constructs regardless of VH-VL domain orientation or linker length, and that long interspace linkers between VH-VL domains are critical for the optimal degranulation and expansion of the CAR T-cells. Because of these findings, we selected the LLH scFv design to proceed with the 3G-CAR cloning, including the ICOS and OX40 ICD. CARs containing the 4-1BB and CD28 were linked to the CD28 TM domain, while CARs with a membrane-proximal ICOS ICD had the ICOS TM domain (unless otherwise indicated), and all the CARs constructs used an IgG4 hinge (**Figure 2A**).

We evaluated three 3G-CAR construct designs [**A.** ROR1-ICOS/4-1BB/CD3ζ (ROR1-IBζ); **B.** ROR1-ICOS/OX40/CD3ζ (ROR1-IOζ); **C.** ROR1-CD28/4-1BB/CD3ζ (ROR1-28Bζ)]; and compared with a 2G-CAR control [ROR1-4-1BB/CD3ζ (ROR1-Bζ)]. There were no differences in the level of CAR protein expression after lentivirus transduction of T-cells (**Supplementary Figure 2**). 3G-CAR T-cells show a statistically significant slower expansion growth than 2G-CAR T-cells (**Figure 2B**). CAR T-cell persistence was evaluated *in vitro* using a model of repetitive target cell challenge developed in our laboratory (49) (**Figure 2D**). On day 1, all the 3G-CAR constructs showed similar E: T ratios suggesting equal cytotoxic potency, and there was a trend for increased cytotoxicity with lower E: T ratios for the cells transduced with the 2G-CAR construct. This trend became statistically significant on Day 2, and by day 5, the cytotoxicity of 2G-CAR T-cells was lost with E:T ratios that were very high and comparable with the untransduced T-cells (UTD). The T-cells transduced with 3G-constructs behave differently; they showed relatively low cytotoxicity during the first 48 hr of culture, and gradually the E:T ratios decreased, and their cytotoxicity was significantly increased by Day 5 compared with the T cells transduced with the 2G-CAR construct or UTD cells (**Figure 2D**). Moreover, after 5 days of *in vitro* rechallenge with ROR1^+^ tumor cell lines, all T cells transduced with the 3G-constructs secreted significantly higher IFN-γ levels than the 2G-CAR construct and UTD control (**Figure 2E**).

In addition, we studied the phenotypic changes of T cells transduced with different constructs, particularly the T-cell central memory (T_CM_) and effector memory (T_EM_) phenotype ratio (T_CM_/T_EM_) that has been associated with CAR T-cell persistence (50). After 12 days of *in vitro* expansion, all the constructs that we tested exhibited at least a threefold increase in CD4 and CD8 T-cell central memory (T_CM_) compared with effector memory (T_EM_) phenotype (**Supplementary Table 3**). However, this elevated “baseline” T_CM_ predominance changed after the cells were evaluated using our five days *in vitro* rechallenge assay with ROR1^+^ tumor cell lines. For example, the 3G ROR1-IOζ CAR T-cells continue to express the highest CD4^+^ T_CM_ levels, with CD8^+^ T_CM_ comparable to the Bζ and IBζ cells and higher than the 28Bζ ones. Regarding the T_EM_ phenotype, the ROR1-IOζ CAR T-cells showed the lowest percentage of T_EM_ cells (CD4^+^ and CD8^+^). The calculated T_CM_/T_EM_ ratios showed a > 4-fold increase in CD4^+^ T-cells transduced with the ROR1-IOζ CAR compared to the other constructs. There were no substantial differences in the T_CM_/T_EM_ ratios among CD8^+^ T cells transduced with any of the constructs (**Figure 2F**; **Supplementary Table 3**).

### 3.3 3G ROR1-IOζ CAR T-cells showed low levels of CD3ζ tonic signaling

We evaluated the tonic signaling of the T cells transduced with different CAR constructs before engagement with the specific target antigen (**Figure 2C**). CAR T cells were expanded for 12 days using T cell media +100 U/ml of IL-2. The baseline CD3ζ CAR chimeric and the native CD3ζ proteins were evaluated for tyrosine phosphorylation (Ty142) by Western blot using specific monoclonal antibodies. This basal CD3ζ signaling helped us to evaluate the tonic phosphorylation of the ICD and potential correlations with cytotoxicity, expansion, and phenotype. CAR T-cells transduced with the 3G ROR1-IOζ showed the lowest phospho-CD3ζ-Ty142 adjusted expression ratios suggesting that this construct induced the lowest ICD tonic signaling activation. On the other hand, T-cells transduced with the 2G ROR1-Bζ showed the highest CD3ζ-Ty142 phosphorylation. Higher phospho-CD3ζ-Ty142 tonic signaling correlated with CAR constructs that induced rapid expansion, cytotoxicity, and exhaustion (**Figure 2**; **Supplementary Figure 4**).

### 3.4 3G-ROR1-IOζ CAR T-cells induce enhanced activity and increased survival in an *in vivo* lymphoma mouse model

**Figure 3:**
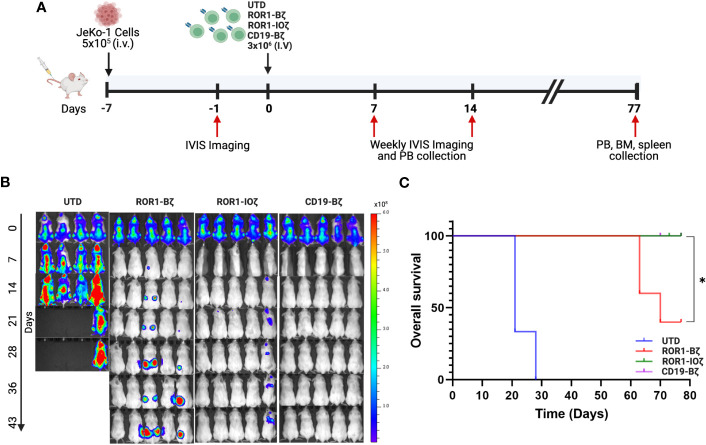
ROR1-10ζ CAR T cells show a long-term durable response and improve overall survival in the mouse model. **(A)** 1 week after the injection of 5 × 10^5^ JeKo-1, mice were randomized into 4 groups: (1) UTD, (2) ROR1-Bζ CART cells, (3) ROR1-10ζ CART cells or (4) CD19-Bζ CART cells. **(B)** Tumor burden was assessed by using BLI (5 mice per group/4 mice per group in UTD). **(C)** Kaplan-Meier survival curves of the ROR1 mouse models treated with UTD, ROR1-Bζ, ROR1-10ζ or CD19-Bζ CART cells. Data are plotted as mean ‡ SEM (* p = < 0.01).

We used the Jeko-1 (ROR1^+^) NHL model in NOD-SCID mice for *in vivo* testing (51). The 3G-ROR1-IOζ construct was selected for these experiments based on the previous results that suggested that this construct could induce better T-cell fitness with a favorable persistent phenotype. We compared the activity *in vivo* of T cells transduced with the 3G-ROR1-IOζ construct *vs*. T cells expressing the 2G-ROR1-Bζ and CD19-Bζ constructs (**Figure 3A**). All of the Jeko-1 tumor-bearing mice were injected with anti-CD19 CAR T-cells, and the 3G-ROR1-IOζ showed excellent response with no clinical evidence of progression (**Supplementary Figure 3**). Three mice injected with the 2G-ROR1-Bζ showed progression and required euthanasia. As expected, the mice treated with untransduced T-cells (UTD) had rapid progression, and all of them died or required euthanasia by Day 28 post-T-cell injection (**Figure 3B**). Kaplan-Meier survival analysis showed that all the mice injected with ROR1-IOζ and CD19-Bζ CAR T-cells survived the 80-day observation study. The median Overall Survival (mOS) of the mice treated with UTD T-cells or ROR1-Bζ CAR T-cells was 20 and 65 days, respectively P< 0.05 (**Figure 3C**). The treated mice developed xenogenic graft-versus-host disease (GVHD), as previously reported on extended CAR T-cell *in vivo* experiments by various groups (52). However, the clinical GVHD manifestations observed in the mice receiving the 2G-ROR1-Bζ T-cells were the most severe.

**Figure 4:**
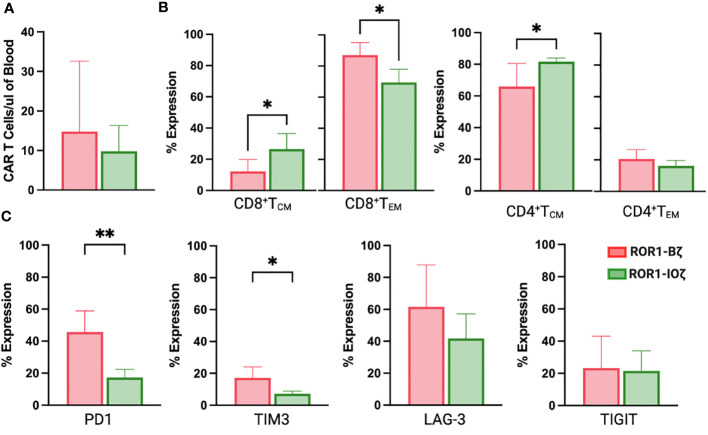
ROR1-10ζ Signaling Domain Leads to Enrichment of Tm Subset, whereas ROR1-B7 Promotes Tem Population. **(A)** Circulating CAR T-cells at day 70 measured by Flow cytometry gating on CD45*CD3*CAR* **(B)**. Relative change of Tem and Tem cell subsets in ROR1-Bζ and ROR1-10ζ CAR T cell cultures. Absolute numbers of live cells were calculated for each population at the specified time points. The graphs show relative fold change of Tm or Tem in ROR1-10ζ CAR T cells normalized to ROR1-Bζ CAR T cells. **(C)** T cell population at day 70 gated on CD3^+^CAR^+^ T cells with exhaustion markers on both groups ROR1-Bζ and ROR1-10ζ Representative plots (from at least 10 mice). Numbers shown are percentages of cells detected in each gate. Absolute number of CART cells per microliter was calculated with counting beads (mean and standard error of the mean). Data are plotted as mean ‡ SEM (**, p< 0.001, and *, p= <0.01).

**Figure 5:**
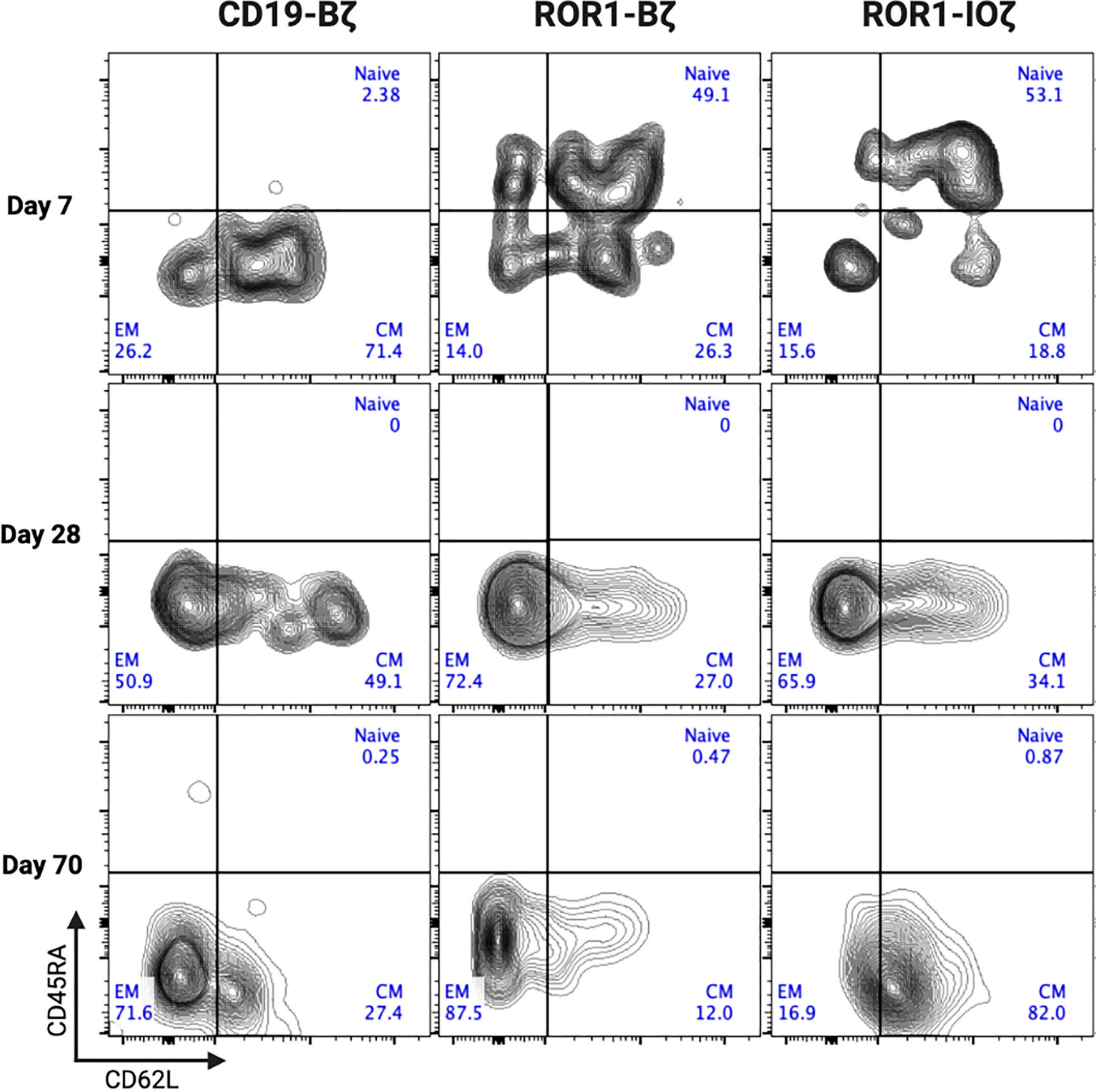
ROR1-IOZ Signaling Domain Leads to Enrichment of Tm Subset, whereas ROR1-B7 Promotes Tem Population. Representative plots (from at least 5 mice) of cell-surface expression of CD62L and CD45RA on CAR T-cells at specified time points during *in vivo* expansion. Cells shown have been pre-gated for live CD3^+^CD8^+^CAR^+^ T cells. Numbers shown are percentages of cells detected in each gate.

We measured circulating anti-ROR1 CAR T-cells in peripheral blood samples collected weekly from Day 0 to Day 70, and we did not observe differences in the median number of cells identified by flow cytometry. For example, median CAR T-cells on Day 70 were 9.8 and 14.8 CAR T cells/μl of blood on ROR1-IOζ and ROR1-Bζ, respectively (**Figure 4A**). We analyze T_CM_, T_EM_ phenotypic profiles (Day 7, Day 28, and Day 70), and exhaustion markers (Day 70) using the same samples. The cells were gated on CD45^+^CD3^+^CAR^+^CD4^+^ for this specific analysis. At Day 70, the circulating CAR T-cells from mice treated with the ROR1-IOζ construct showed a significantly higher number of T_CM_ and lower number of T_EM_ cells than ROR1-Bζ (**Figure 4B**). In addition, the CAR T-cells from the group of mice treated with the 3G-ROR1-IOζ T-cells, have low expression of exhaustion markers, including PD1, LAG3, TIGIT, and TIM-3 (**Figure 4C**). CD8^+^ T-cells expressing central memory phenotype corresponded to 82% of the total population by Day 70 in the mice treated with 3G-ROR1-IOζ CAR T-cells compared to only 12% on those receiving the 2G-ROR1-Bζ similar distribution observed in the 2G-CD19-Bζ (**Figure 5**).

## 4 Discussion

CAR T-cells’ proliferation, expansion, and persistence correlate directly with the clinical response to B cell malignancies (6, 53, 54). Unfortunately, these optimal CAR T-cell characteristics have not been observed in clinical trials of patients with solid tumors (1, 4–6). Therefore, a deeper understanding of the factors that enhance proliferation, expansion, and persistence will be of significant importance in improving the treatment outcomes of cancer patients treated with CAR T-cell therapies.

Multiple strategies have been investigated to increase the persistence of CAR T-cells, specifically cells with a T_CM_ phenotype. Some of those include patient vaccination to enhance tumor-reactive clones and perform a selection of those prior to *ex vivo* manipulation, optimization of CAR T-cell culture conditions, carefully monitoring electrolyte, amino acids, carbohydrates, and cytokines content with the goal to increase T-cell naïve (T_N_) and T_CM_ (55–59), modification of metabolic pathways of the CAR T-cells that reduced the glycolytic activity and increase fatty acid oxidation (FAO). This step is critical for T_CM_ generation (60) or direct engineering of the CAR using modifications, particularly in the ICD regions, to enhance cytotoxicity and persistence (15–17, 21, 61, 62). In the work presented here, we have evaluated this latter strategy by studying the activity of ICOS and OX40 by creating a novel tandem ICD that could have broad applications in CAR T-cell design.

We selected ROR1 as the molecular target of our experiments because ROR1 is a TAA expressed in a wide variety of cancers (35–39); therefore, any optimization derived from our work will have a broad range of applications. In our initial experiments, we observed that the positional sequence of VH-VL domains of the scFv did not have a significant impact on the level of CAR expression but appears to play a role in facilitating CAR T-cell expansion, mainly when the VH domain follows the VL domain with a long linker G4S_(3)_ (LLH 5’-3’ cloning sequence). Similarly, our data show that a long linker increased degranulation evaluated with the expression of CD107a. Our observations are consistent with previous reports describing the importance of VH and VL positional cloning and other elements like peptide and non-peptide linkers associated with the scFv design. The orientation of the domains and the spatial/tridimensional configuration facilitated by the spacer linker can impact the stability, specificity, and activity of the scFv molecule (63–66). Whether or not these “rules” are universal or if it is required to optimize each scFv for specific applications individually is something that needs to be clarified in future studies.

Using the LLH cloning sequence as the CAR scaffold design, we tested the activity of ICD that included ICOS and OX40. Our data show that 3G CAR construct designs with two domains linked to the CD3ζ motif tend to grow slower, but at the same time, they exhibit higher levels of targeted cytotoxicity for more extended periods, as demonstrated in our five days repeat challenge stimulation. The 2G CAR construct we used as a control, ROR1-Bζ, induced high levels of T-cell cytotoxicity but after three days, that activity decreased significantly and, by day five, was comparable with the untransduced (UTD) cells. The rapid decay of the cytotoxic activity of the 2G-CAR construct was most likely due to the initial ICD medicated overactivation, followed by a gradual increase in the expression of exhaustion markers, as shown previously (67–69). We placed ICOS upstream in the CAR sequence, close to the membrane, as it has been demonstrated that this is critical for optimal signaling of this ICD (20). In our experiments, the 3G CAR construct using CD28 and 4-1BB together (ROR1-28Bζ) showed a mixed cytotoxic profile with potent cytotoxicity early and maintained until the end of the five days of incubation. However, T-cells transduced with the ROR1-IOζ construct showed an uniform immunological fitness profile with high levels of persistent cytotoxicity associated with high IFN-γ production, T_CM_ polarization, particularly in CD4^+^ T-cells and a high T_CM_/T_EM_ ratio. Our study focused primarily on the specific T-cell phenotypic profiles induced by CAR ICDs and their correlation with cytotoxicity, cytokine production, persistence, and central memory phenotype. Another potential explanation for these findings could be the ICD-induced tonic signaling (69, 70). Recent studies have shown that optimal tonic signaling correlates with CAR T cell expansion, performance, and exhaustion profile (71). CAR construct tonic signaling provides a basal activation level necessary for an efficient engagement with the antigen expressed in the target cell (71). In addition, CAR tonic signaling contributes to maintaining CAR-T cell viability, promoting memory-like properties, and enhancing their overall antitumor activity (72).

ICOS (Inducible T cell co-stimulator) and OX40 (TNFRSF4 – CD134) are costimulatory receptors that play a crucial role in developing central memory T cells and their persistence. Central memory T cells are a subpopulation essential for rapid and effective responses to subsequent infections or tumoral challenges (50, 73). ICOS and OX40 help to enhance T cells’ activation, expansion, and survival, leading to the development of a strong and durable memory T cell response and T_CM_ long-term protective immunity pool (20). In addition, the activation of ICOS and OX40 can enhance cytokine production and effector function, further increasing the effectiveness of T-cell responses and inducing a Th1, Th17 T-cell polarization (32). OX40 can enhance Tregs activation in autoimmune disease, and this is a potential adverse event that will need to be assessed in future clinical development of constructs bearing the OX40 ICD (74, 75). On the other hand, the activation of OX40 can trigger additional anti-tumoral signaling promoting Bcl-xL and Bcl-2 expression, essential for long-term T-cell survival (76) and elimination of the tumor suppressive activity of regulatory FOXP3+CD25+CD4+ T cells (77).

We observed the effect of the ICOS-OX40 combination *in vivo*. Jeko-1 tumor-bearing mice showed a significant response after injection of ROR1-IOζ CAR T-cells. They showed rapid tumor responses, the best O.S. superior to 2G-CAR ROR1-Bζ. We excluded the possibility that the antitumor effect of the ROR1 CARs resulted from their allogeneic effect because the UTD cells did not show any evident effects on tumor growth. Assessment of T cell phenotype at the end of the study on Day 70 showed that ROR1-IOζ CAR T-cells have an elevated T_CM_/T_EM_ ratio, progressive accumulation of T_CM,_ and lower expression of exhaustion markers. In addition, we observed that the initial expansion process of the T-cells induced elevated T_CM_/T_EM_ ratios before *in vivo* or *in vitro* testing, regardless of transduction with CAR constructs (**Supplementary Figure 2**). This suggests that the initial phenotypic characteristics of the product did not influence the T_CM_ polarization observed over time after the CAR T-cells encountered the target. We understand that NSG mouse model does not provide any information related with the tumor microenvironment due to the lack of immune system but can serve as suitable model for CAR T antitumor activity studies (78, 79). These data strongly indicate that the ICD modifications of the ROR1-IOζ CAR construct confer a T-cell immunological fitness profile that favors cellular persistence (43, 50, 80). Our findings provide guidance for the synthetic design and optimization of CAR T-cells with enhanced persistence, which is a desirable characteristic of highly effective immune effector cells for cellular therapy. Moreover, we proved evidence that ICOS and OX40 assembled in a 3G-CAR T-cell configuration induces phenotypic changes that favor T_CM_ phenotype and enhance *in vivo* and *in vitro* redirected cytotoxicity. The novel combination of tandem ICD ICOS-OX40-CD3ζ described here, represents an alternative for broad applications in CAR design and cellular immunotherapy.

## Data availability statement

The original contributions presented in the study are included in the article/**Supplementary Material**. Further inquiries can be directed to the corresponding author.

## Ethics statement

Ethical approval was not required for the studies on humans in accordance with the local legislation and institutional requirements because only commercially available established cell lines were used. The animal study was approved by The Mayo Clinic Hospital Institutional Animal Care and Use Committee. The study was conducted in accordance with the local legislation and institutional requirements.

## Author contributions

EM-C, JF, and JC designed the study and planned the experiments. EM-C, JF, JG-R, and NB performed the experiments. EM-C, JF, JG-R, PF-F, and JC analyzed the data. EM-C, JF, JC. Designed the constructs and generated plasmids. EM-C and PF-F performed animal experiments. EM-C and JC wrote the manuscript. EM-C, PF-F, JC, JF edited the manuscript.
