# Supplementary material for: ICOS and OX40 tandem co-stimulation enhances CAR T-cell cytotoxicity and promotes T-cell persistence phenotype
Source: Front Oncol. 2023 Aug 18;13:1200914. doi: 10.3389/fonc.2023.1200914 (PMC10502212; doi:10.3389/fonc.2023.1200914)
Supplement: Supplementary file 1 [file Presentation_1.pptx]

## Slide 1
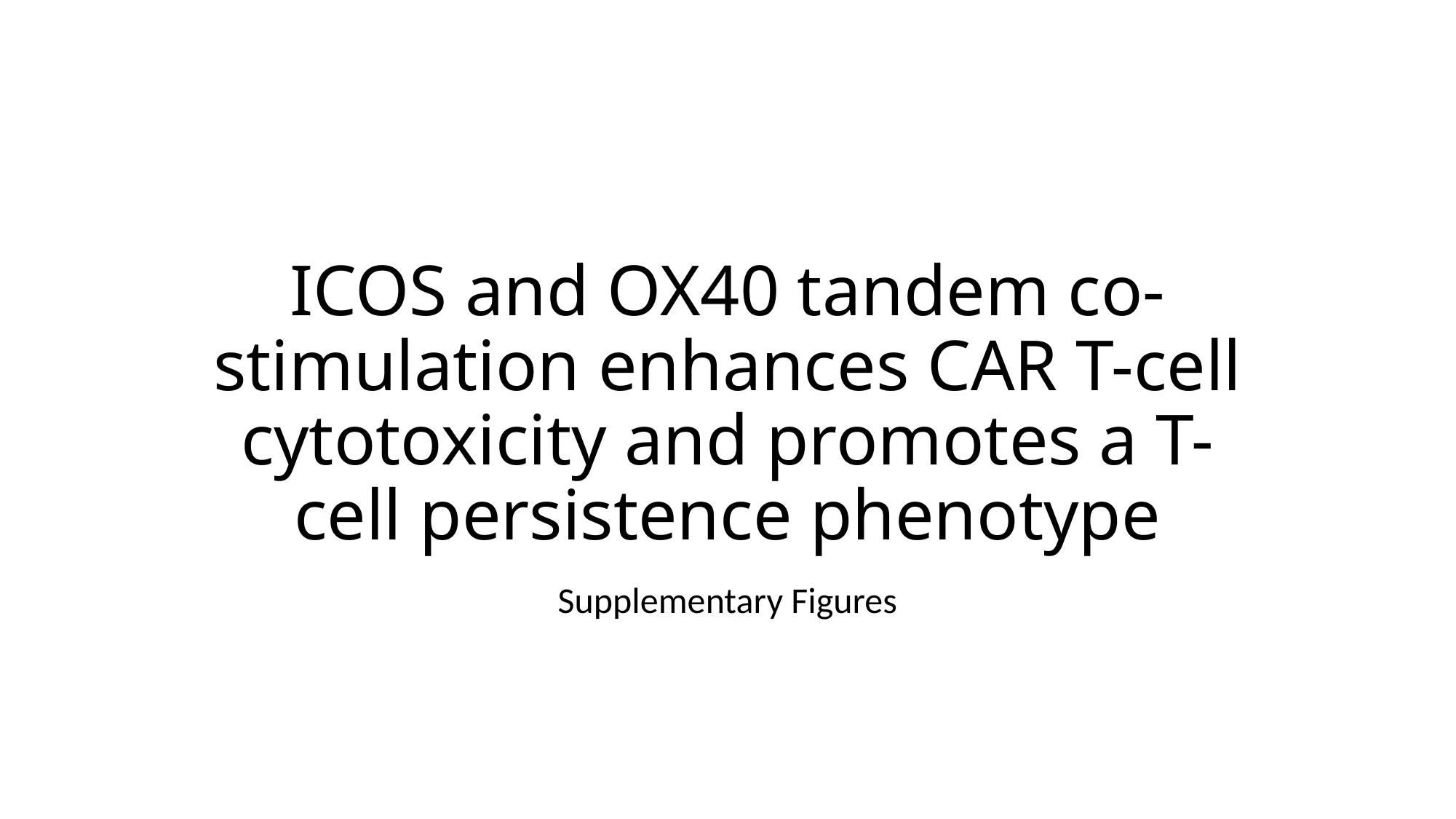

# ICOS and OX40 tandem co-stimulation enhances CAR T-cell cytotoxicity and promotes a T-cell persistence phenotype
Supplementary Figures

## Slide 2
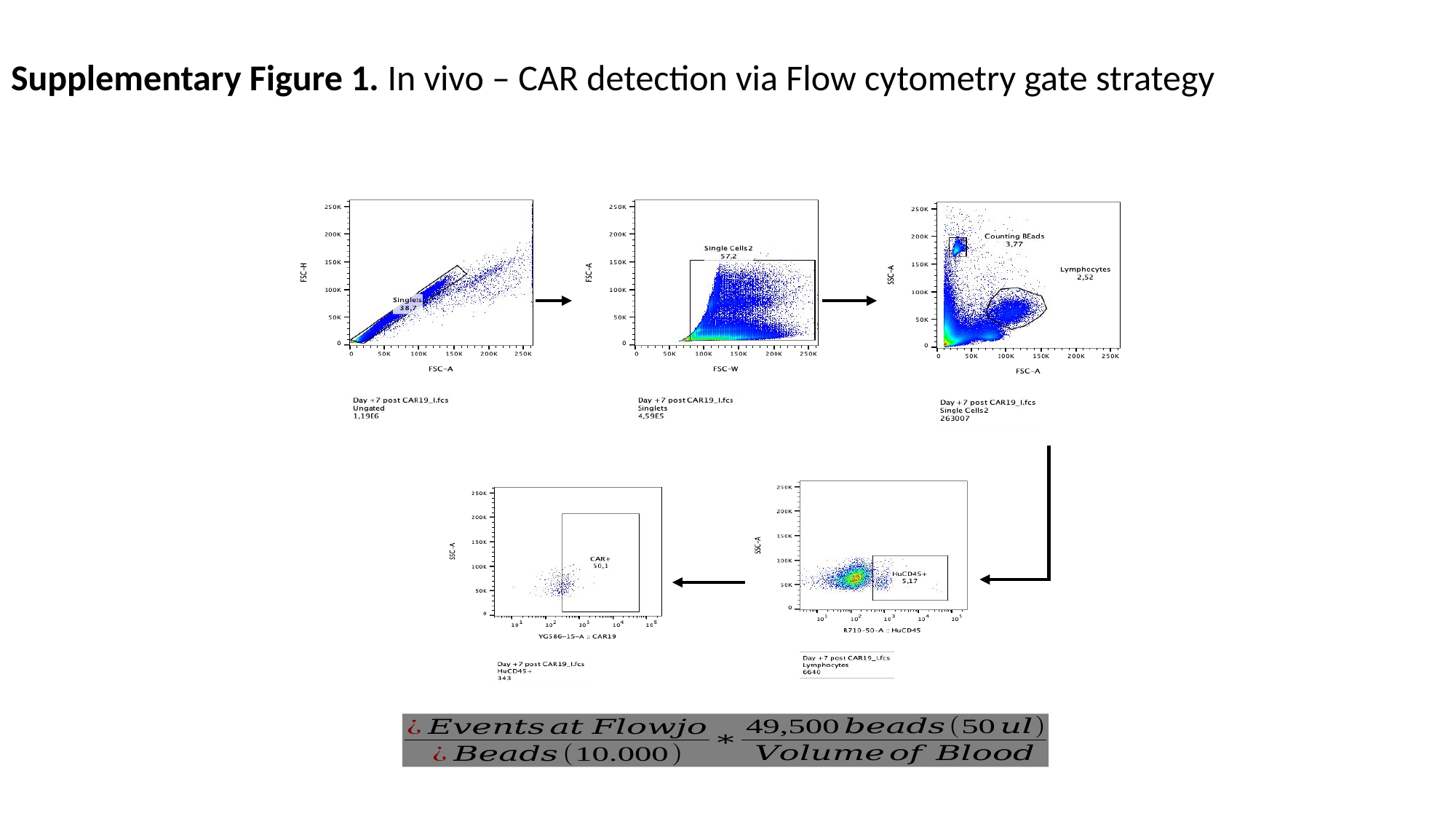

Supplementary Figure 1. In vivo – CAR detection via Flow cytometry gate strategy

## Slide 3
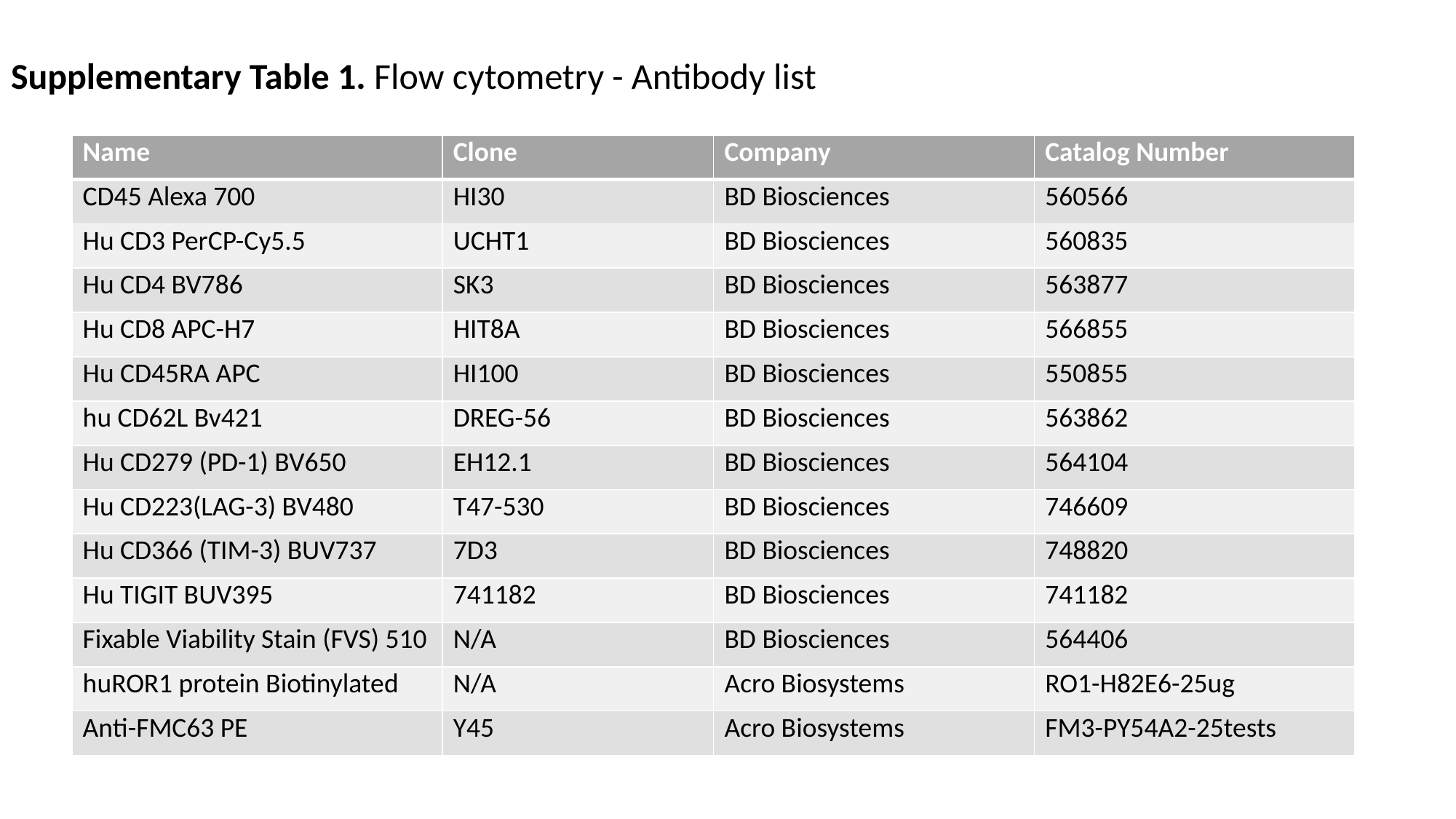

Supplementary Table 1. Flow cytometry - Antibody list
| Name | Clone | Company | Catalog Number |
| --- | --- | --- | --- |
| CD45 Alexa 700 | HI30 | BD Biosciences | 560566 |
| Hu CD3 PerCP-Cy5.5 | UCHT1 | BD Biosciences | 560835 |
| Hu CD4 BV786 | SK3 | BD Biosciences | 563877 |
| Hu CD8 APC-H7 | HIT8A | BD Biosciences | 566855 |
| Hu CD45RA APC | HI100 | BD Biosciences | 550855 |
| hu CD62L Bv421 | DREG-56 | BD Biosciences | 563862 |
| Hu CD279 (PD-1) BV650 | EH12.1 | BD Biosciences | 564104 |
| Hu CD223(LAG-3) BV480 | T47-530 | BD Biosciences | 746609 |
| Hu CD366 (TIM-3) BUV737 | 7D3 | BD Biosciences | 748820 |
| Hu TIGIT BUV395 | 741182 | BD Biosciences | 741182 |
| Fixable Viability Stain (FVS) 510 | N/A | BD Biosciences | 564406 |
| huROR1 protein Biotinylated | N/A | Acro Biosystems | RO1-H82E6-25ug |
| Anti-FMC63 PE | Y45 | Acro Biosystems | FM3-PY54A2-25tests |

## Slide 4
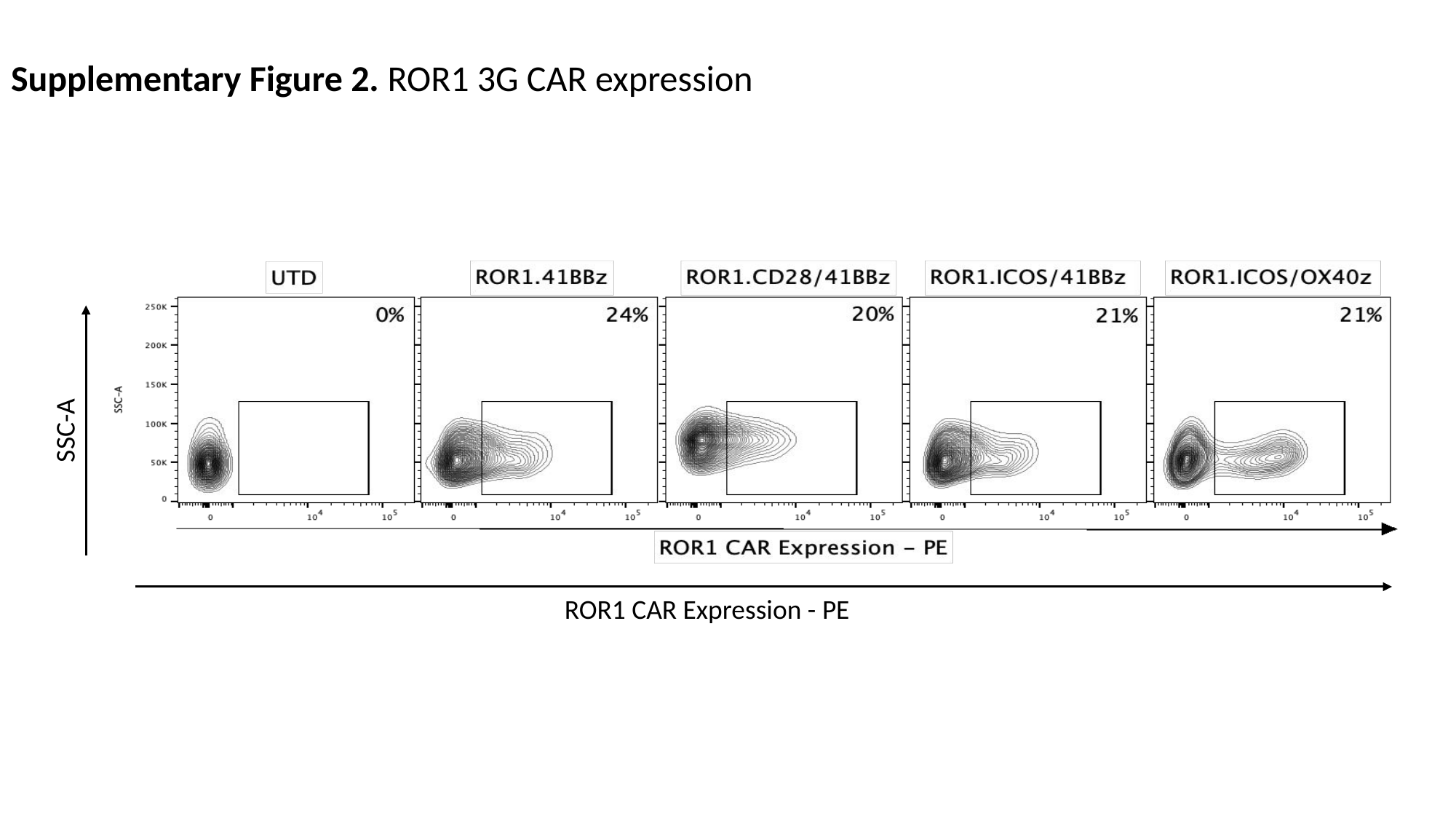

# Supplementary Figure 2. ROR1 3G CAR expression
SSC-A
ROR1 CAR Expression - PE

## Slide 5
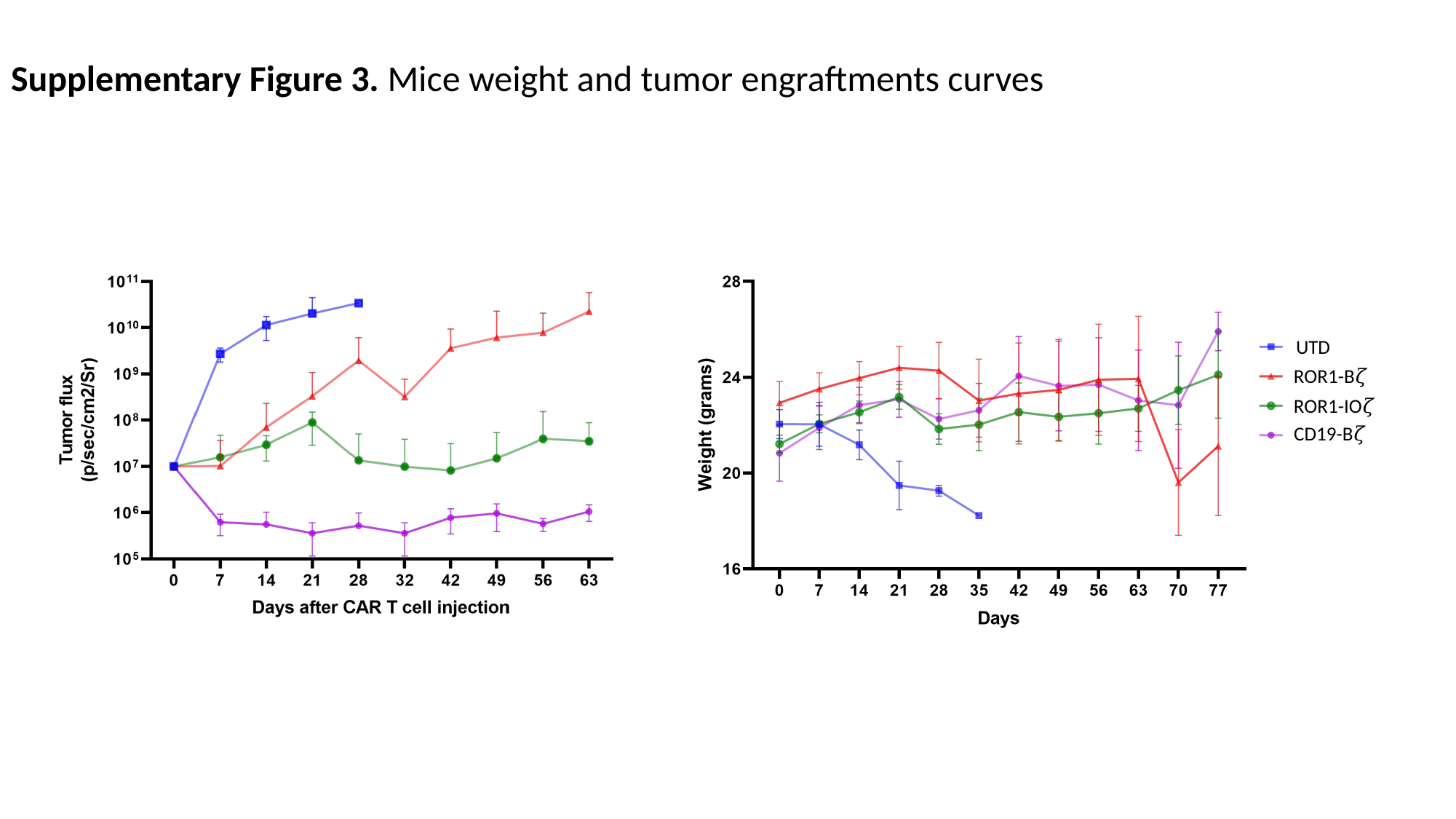

# Supplementary Figure 3. Mice weight and tumor engraftments curves
UTD
ROR1-B𝜁
ROR1-IO𝜁
CD19-B𝜁

## Slide 6
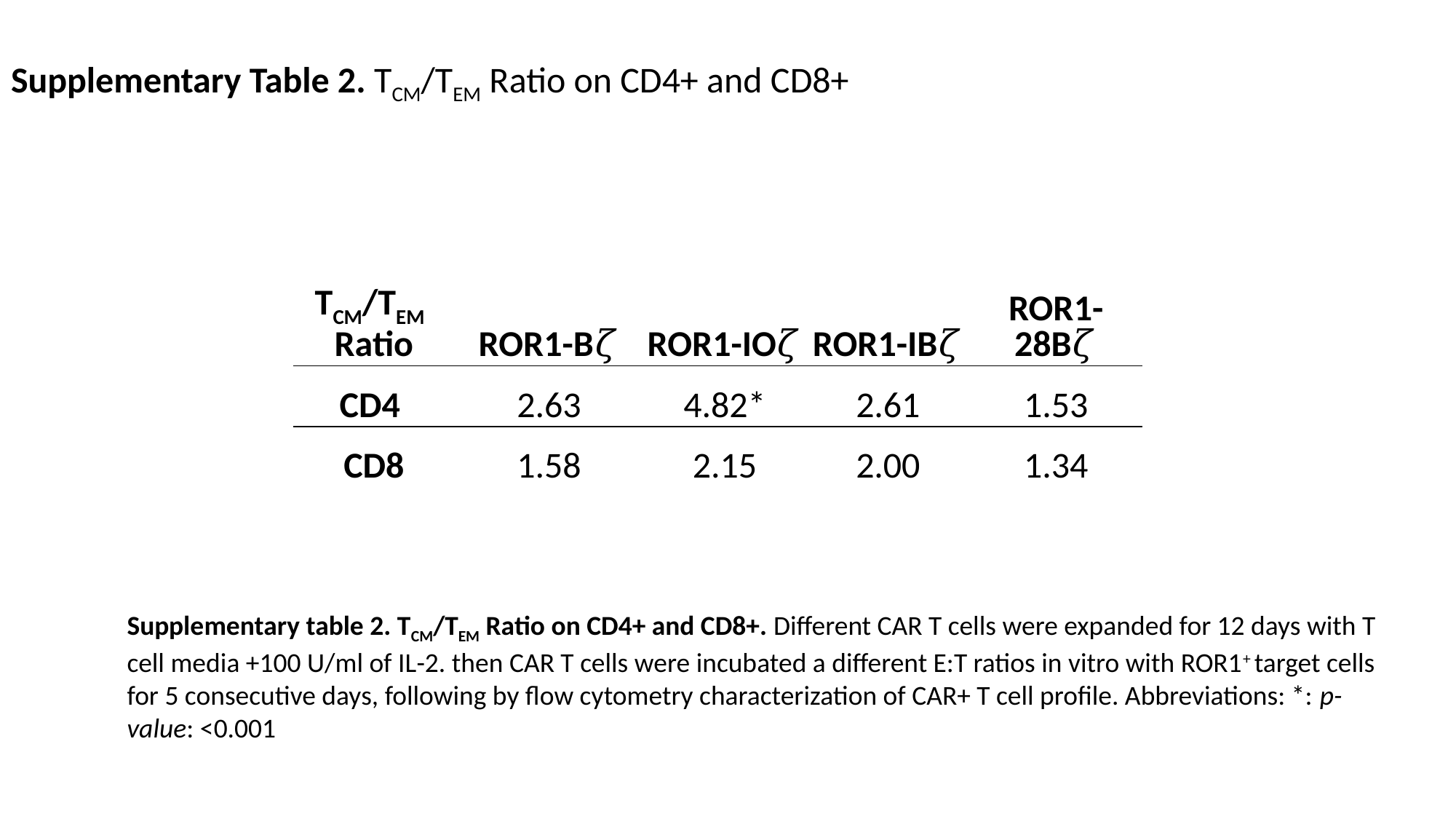

# Supplementary Table 2. TCM/TEM Ratio on CD4+ and CD8+
| TCM/TEM Ratio | ROR1-B𝜁 | ROR1-IO𝜁 | ROR1-IB𝜁 | ROR1-28B𝜁 |
| --- | --- | --- | --- | --- |
| CD4 | 2.63 | 4.82\* | 2.61 | 1.53 |
| CD8 | 1.58 | 2.15 | 2.00 | 1.34 |
Supplementary table 2. TCM/TEM Ratio on CD4+ and CD8+. Different CAR T cells were expanded for 12 days with T cell media +100 U/ml of IL-2. then CAR T cells were incubated a different E:T ratios in vitro with ROR1+ target cells for 5 consecutive days, following by flow cytometry characterization of CAR+ T cell profile. Abbreviations: *: p-value: <0.001

## Slide 7
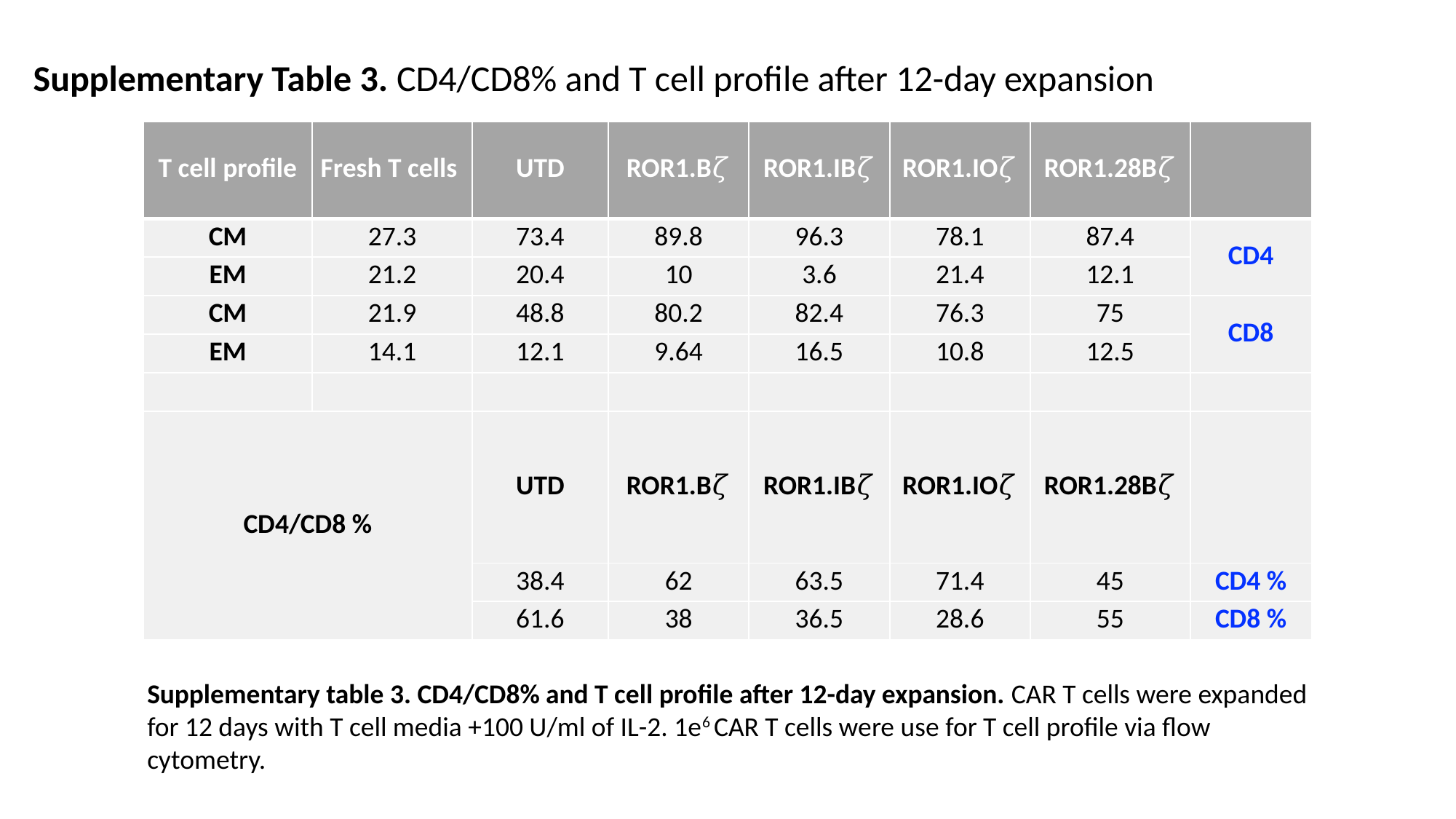

# Supplementary Table 3. CD4/CD8% and T cell profile after 12-day expansion
| T cell profile | Fresh T cells | UTD | ROR1.B𝜁 | ROR1.IB𝜁 | ROR1.IO𝜁 | ROR1.28B𝜁 | |
| --- | --- | --- | --- | --- | --- | --- | --- |
| CM | 27.3 | 73.4 | 89.8 | 96.3 | 78.1 | 87.4 | CD4 |
| EM | 21.2 | 20.4 | 10 | 3.6 | 21.4 | 12.1 | |
| CM | 21.9 | 48.8 | 80.2 | 82.4 | 76.3 | 75 | CD8 |
| EM | 14.1 | 12.1 | 9.64 | 16.5 | 10.8 | 12.5 | |
| | | | | | | | |
| CD4/CD8 % | | UTD | ROR1.B𝜁 | ROR1.IB𝜁 | ROR1.IO𝜁 | ROR1.28B𝜁 | |
| | | 38.4 | 62 | 63.5 | 71.4 | 45 | CD4 % |
| | | 61.6 | 38 | 36.5 | 28.6 | 55 | CD8 % |
Supplementary table 3. CD4/CD8% and T cell profile after 12-day expansion. CAR T cells were expanded for 12 days with T cell media +100 U/ml of IL-2. 1e6 CAR T cells were use for T cell profile via flow cytometry.

## Slide 8
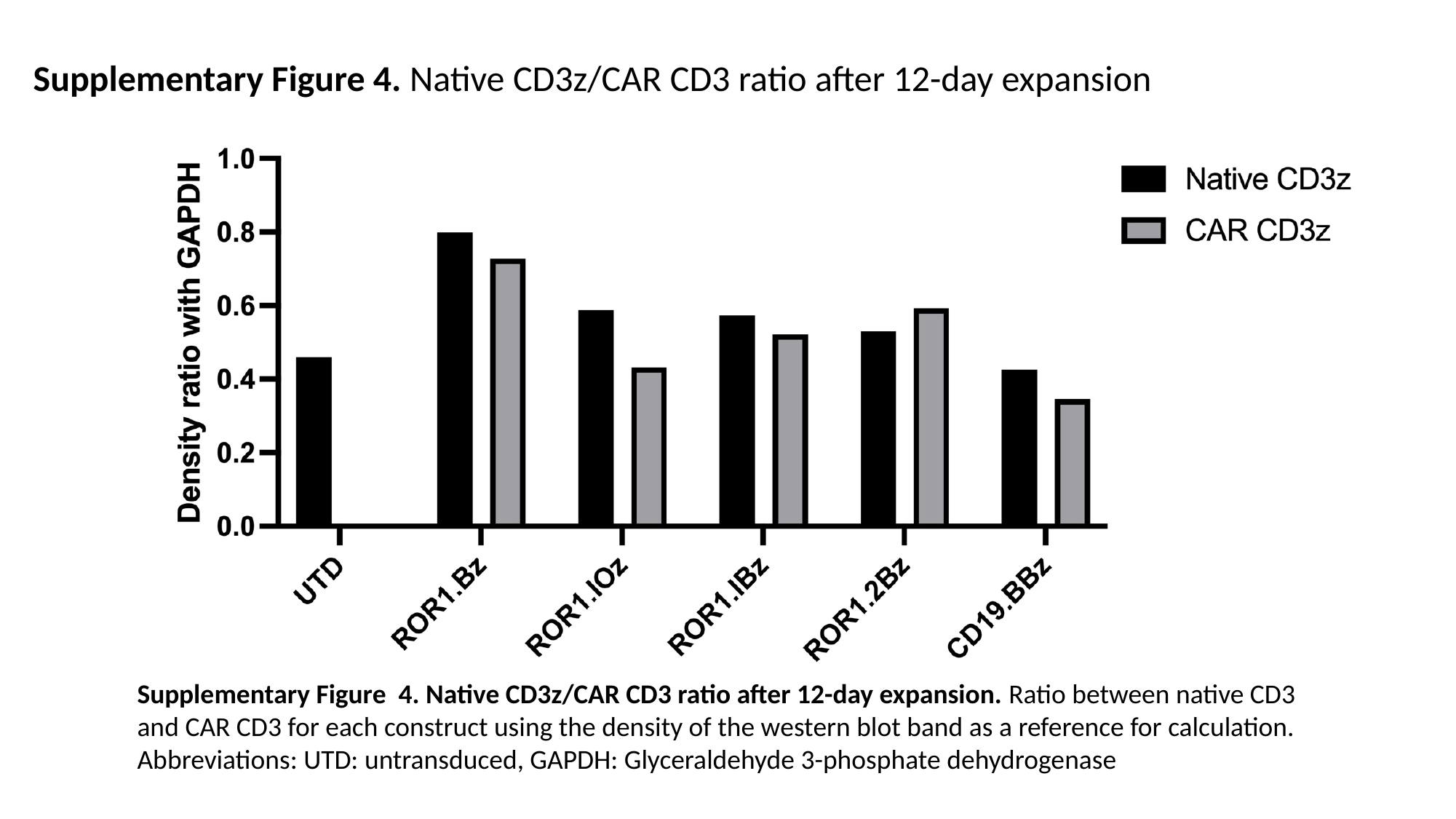

# Supplementary Figure 4. Native CD3z/CAR CD3 ratio after 12-day expansion
Supplementary Figure 4. Native CD3z/CAR CD3 ratio after 12-day expansion. Ratio between native CD3 and CAR CD3 for each construct using the density of the western blot band as a reference for calculation. Abbreviations: UTD: untransduced, GAPDH: Glyceraldehyde 3-phosphate dehydrogenase
